# Extreme Risk-Taking Behaviors in Patients With Eating Disorders

**DOI:** 10.3389/fpsyt.2020.00089

**Published:** 2020-02-28

**Authors:** Daniel Stein, Shikma Keller, Inbar Sharav Ifergan, Tal Shilton, Anat Toledano, Maya Treves Pelleg, Eliezer Witztum

**Affiliations:** ^1^ Pediatric Psychosomatic Department, Safra Children's Hospital, Tel Hashomer, Israel; ^2^ Sacker Faculty of Medicine, Tel Aviv University, Tel Aviv, Israel; ^3^ Department of Psychiatry, Hadassah University Medical Center, Jerusalem, Israel; ^4^ Eating Disorders Center, Sheba Medical Center, Tel Hashomer, Israel; ^5^ Faculty of Health Sciences, Division of Psychiatry, Ben Gurion University of the Negev, Beer Sheva, Israel

**Keywords:** anorexia nervosa, attempted suicide, non-suicidal self-injury, self-harm, suicide

## Abstract

**Background:**

Patients with eating disorders (EDs) engage in different self-inflicted at-risk behaviors, including suicide, attempted suicide and non-suicidal self-injury. Our aim was to describe the occurrence and underlying motivations of non-suicidal extreme risk-taking behaviors in patients with EDs.

**Methods:**

Four cases from different treatment centers in Israel were analyzed.

**Results:**

All patients were females hospitalized in inpatient settings because of long lasting anorexia nervosa (AN) with either binge/purge or purging episodes (AN-B/P/AN-P), including in most cases both self-induced voting and laxative abuse. Case [1] was an adolescent also diagnosed with type 1 diabetes mellitus. She abused insulin, both omission and overdose, was highly suicidal, and suffered from comorbid oppositional behavior, depression and anxiety. Case [2] was a 24-years old woman, transitioning from restricting to AN with vomiting and laxative use during inpatient treatment. She was also diagnosed with attention deficit hyperactivity disorder, depression, anxiety, and suicidal thoughts. In hospital, she developed excessive water consumption, leading to very low urine concentrations and sodium levels, and one episode of loss of consciousness. Case [3] was in her late thirties, demonstrating particularly massive laxative abuse. She also suffered from alcohol addiction, sexual trauma, and one attempted suicide. During hospitalization she developed laxative-abuse-related rectal prolapse that was successfully operated. Nonetheless, after operation she resumed laxative abuse. Case [4] was a 23-year old pregnant women with highly active AN-B/P during pregnancy. She was hospitalized at 23 weeks of gestation following abdominal pressure. She only partly complied with inpatient treatment, discharged herself against medical advice after 5 weeks, and gave birth at week 34.

**Discussion:**

All cases were females with long-standing B/P type AN, often with multiple purging behaviors, other impulsive and non-impulsive comorbidities, and many environmental vulnerabilities. Different motivations were found for these extreme behaviors in addition to ED-related factors, mostly not related to suicide. The severity of the medical and psychological condition required multimodal medical and psychological inpatient interventions. The patients mostly did not comply with their treatment, showing considerable indifference to their grave medical condition.

## Introduction

Eating disorders (EDs) show a lifetime prevalence of around 4% for DSM-5 diagnoses of anorexia nervosa (AN), and 2% each for bulimia nervosa (BN), and binge eating disorder (BED) (1, 2). AN, BN, and BED are associated with numerous physical (e.g., osteoporosis, fluid electrolyte imbalance, and metabolic syndrome) and comorbid mental health (e.g., affective and anxiety disorders), conditions (1, 3). Actually, the medical complications caused by EDs may involve almost all organ systems and can be viewed as consequences of: undernutrition, purging behaviors, and binge eating. The medical complications of EDs are significant and potentially life-endangering and irreversible (4). Moreover, medical complications may account for more than half of all deaths in patents with AN (5). Last, patients with all types of EDs have significantly elevated mortality rates compared with the standard population norms (3, 6). Actually, AN is associated with the highest rate of mortality among all mental disorders (7).

### Dangerous Behaviors in Patients With EDs

Patients with EDs are at a high risk for endangering their life and overall health. Suicide is likely the most dangerous behaviors, being particularly frequent in AN (3). Actually, AN is considered the mental health disorder with the highest suicide rates (8). Completed suicide is less frequent in BN than in AN (3), and highly infrequent in BED (9).

In contrast to completed suicide, attempted suicide is more frequent in patients with BN than AN, and in AN of the binge/purging type (AN-B/P) compared with restrictive-type AN (AN-R) (8). The frequency of attempted suicide and the severity of the medical threat of the attempts in AN and BN are comparable to those found in major depression and conduct disorder, and greater than those found in schizophrenia and anxiety disorders (10). Attempted suicide in EDs is particularly elevated in the context of impulsivity (8).

Many factors have been associated with the increased suicide risk in AN. Disturbances in body image and body dissatisfaction represent one major factor for elevated suicidal risk in patients with EDs (11). Nonetheless, according to 12, it is not merely the fear of gaining weight and the pursuit of thinness that increase suicidality in EDs. Thus, incorrect interpretation of interoceptive stimuli, insensitivity to bodily functions, and lack of bodily control may eventuate in a detachment from the body, leading, in turn, to self-neglect and facilitation of self-destructive behaviors (12).

Another dangerous self-destructive behavior in EDs is non-suicidal self-injury (NSSI). This is a direct and deliberate destruction of one's bodily tissue without suicidal intent. Although NSSI and suicidal behavior (completed and attempted suicide) are distinct in many ways, including their primary motivation, the method employed in the self-destructive behavior, and the medical severity involved in each, the presence of NSSI is associated with an increased risk of suicide (1, 13).

Up to 68% of patients with EDs may engage in NSSI, and between 25% and 54% of people who engage in NSSI report comorbid disordered eating (14). Similar to attempted suicide, NSSI is associated with a greater risk of B/P pathology (15, 16) and elevated impulsivity and emotion dysregulation (8, 16, 17). Self-injurious patients with EDs may in addition represent with more dysfunctional personality traits, particularly, higher levels of harm-avoidance and lower levels of self-directedness (17).

In general, NSSI, including in EDs, may be understood as a means to adapt to inner and outer demands (18). Several models, potentially accounting for the occurrence of NSSIs in mental disorders, including in EDs, have been proposed (18, 19):

The affect regulation model regards NSSI as a maladaptive emotion regulation strategy, designed to prevent the escalation of negative affects such as anxiety, or, alternatively, to evoke emotions when feeling numb or dissociated (8, 16, 18, 19). Indeed, NSSI in ED patients may be preceded by high-activation unstable affective states (20). Moreover, the regulatory character of this strategy is suggested by the self-injurers' claim that the sight of blood, or alternatively the sense of physical pain (against detachment or mental pain), helps them to calm down.

Second, the environmental model focuses on the behavior learning process and its power to reinforce NSSI, viewing it as socially learned behavior, employed to communicate, manipulate, or influence one's immediate environment. The contention that individuals who self-injure have poor communication and social skills (18) is of particular relevance in patients with AN, who may present with elevated levels of autistic features and inability to identify and communicate thoughts, emotions, and sensations (21).

Third, the self-punishment model relates to the high prevalence of childhood maltreatment (abuse and neglect) found among individuals who self-injure (18), including in patients with EDs, as well as to the guilt feelings of patients with AN that the place they occupy in the world is at the cost of others (22). Last, the interpersonal boundaries model stipulates that individuals who self-injure are not only likely to have suffered childhood maltreatment, but have also been separated from primary caregivers in early years—whether due to divorce, death, or parental neglect. Such losses of early important objects are highly prevalent in patients with EDs (23). Consistent with these findings, self-injurers may fail to achieve sufficient levels of self and object-representation differentiation, leading them to experience the loss of others as if it is loss of their own self (18).

Other motivations may also increase the risk of endangering behaviors in patients with EDs. These include indifference to what might happen with their immediate life (24), owing, perhaps, to reduced attraction to life (25), detachment from one's body because of reduced interoceptive sensation awareness (21), reduction of emotional involvement (16, 21), and the greater likelihood of inhibited over-controlled affective responses in patients with AN (26).

To summarize, ED patients with concurrent NSSI behaviors may have psychopathological, cognitive, and emotional characteristics that differ from patients with EDs only (8, 17).

## Case Descriptions

The present article describes four women with AN developing different extreme at-risk behaviors during the course of their illness. An attempt will be made to summarize common as well as specific aspects of these behaviors. Written informed consents have been obtained from the participants (and from the parents of one participant who was younger than 18) for the publication of these case reports.

Three cases of the four described here suffered from different medical derangements that were either a direct result of the ED, or appeared in the context of the ED: These include comorbid type 1 diabetes mellitus (T1DM) and AN, psychogenic polydipsia in a patient with AN, and rectal prolapse resulting from massive laxative abuse in another patient with AN. The fourth patient suffered from active AN during her pregnancy.

Regarding the development of AN in young people with T1DM, as shown in case [1], each disorder may adversely influence the course of the other. Malnutrition in AN may lead to hypoglycemia and increase insulin resistance, whereas insulin administration may lead to increase in weight due to increased metabolic efficiency, likely worsening the ED (27). The contrasting weight fluctuations associated with T1DM (reduction in weight) and insulin treatment (increase in weight) represent a considerable burden for girls with AN (28), increasing their body dissatisfaction, weight preoccupation and drive to lose weight (29). Hence, adolescents with comorbid AN and T1DM show considerable difficulties in handling their illness (30), and their compliance and adherence with their treatment is limited (31).

Psychogenic polydipsia, described in case [2], is characterized by excessive oral fluid intake in the absence of physiologic stimuli to drink, or any underlying related organic disease (32). It may represent a potentially at-risk condition, associated with severe hyponatremia and hypokalemia, potentially culminating in loss of consciousness, convulsions, and cardiac arrythmias. Although psychogenic polydipsia is common in patients with AN, and only rarely leads to serious clinical complications (24, 33, 34), if such complications do occur, as shown in case [2], they might become highly dangerous (24, 33–40).

The most frequent motivations associated with psychogenic polydipsia in patients with AN include intentional falsifying of low weight in routine weight measurements, a form of “purging behavior” to purify the body with diuresis, and as a means to reduce calories, suppress hunger, drink instead of eating, and induce vomiting (24, 33, 35, 39). Last, it might also represent a variant form of BN (35, 40).

As shown in case [3], patients with EDs use laxatives to get rid of calories and lose weight. In other cases, laxative abuse may be synonym to other maladaptive self-harm self-soothing behaviors such as cutting (8). Over time, the intestines build up resistance to laxatives, hence dosages must be increased to obtain the same result. The renin-aldosterone system is activated by the massive fluid loss; consequently, when laxative use is discontinued, edema and rapid weight gain occur because of fluid retention. Feelings of fullness and weight gain cause intense anxiety for patients with EDs, further reinforcing laxative abuse.

Laxative abuse may occur in 10%–60% of patients with EDs (41–43). Changes in electrolyte and acid-base balance associated with laxative misuse, mainly metabolic acidosis and hypokalemia, may involve the cardiovascular system and kidney, and are potentially life-threatening (41, 44). Chronic laxative use can cause irreversible damage to the smooth muscles comprising the intestine (41), inducing in rare cases, as shown in our patient, rectal prolapse, where the entire bowel wall protrudes from the anus (45–47). Factors increasing the risk of rectal prolapse in patients with EDs include recurrent bingeing behaviors, constipation, laxative abuse, intense effort during physical exercise, and increase in intra-abdominal pressure related to intentional vomiting or to prolonged periods of sitting on the toilet during defecation (42, 47).

The fourth patient shows a different type of ED-related at-risk behavior, i.e., endangering the course and outcome of pregnancy occurring during active AN. Until relatively recently, the occurrence of pregnancy in women with AN would have been considered inconceivable—both psychologically and physiologically (48). However, in the past decade, there is a significant increase in the frequency of pregnancy among active AN patients (48, 49), mainly because of greater accessibility to fertility treatments (49).

Many problems may stem from the occurrence of pregnancy in women with AN, first and foremost, that pregnancy and low weight and a flat stomach cannot coexist. Some women fail to make the choice of having a baby over losing their perfect AN body. Others might go to extremes in their desire to have both, even at the cost of possibly endangering themselves and/or their offspring.

### Case 1: Anorexia Nervosa and Type 1 Diabetes Mellitus: the Oxymoron of Comorbidities

L. is 17.8 years old, the oldest of three girls, with T1DM, celiac disease and lactose sensitivity. She was hospitalized in a specialized inpatient ED department at the age of 16.3 because of AN.

L. was described from an early age as having a hard temperament and was treated in a child developmental clinic because of communication difficulties. She had social difficulties since early age, being introverted and shy, and having minimal contact with her peers. Her parents described in addition oppositional behavior at home, followed by temper outbursts and family conflicts.

At the age of 13, L. was diagnosed with celiac disease, having to change her eating regimen. She had difficulties in maintaining her weight and experienced secondary amenorrhea. In a psychiatric consultation, she was diagnosed with social anxiety disorder and a depressive episode and was treated with cognitive behavioral treatment. Her relationship with her parents deteriorated to constant control-struggles, followed by long periods of no communication.

Later, L. developed T1DM. Insulin therapy was initiated, and L. started to gain weight, achieving menstrual stability. She was very unhappy with her weight gain, exhibiting severe restrictive behavior, followed by self-induced vomiting and laxative abuse.

L. was brought to an emergency room when the reduction of food intake culminated in an almost complete starvation. She was hospitalized and diagnosed with purging disorder. When her physical condition stabilized, L. was admitted to an ED daycare program. As she did not cooperate with this program. L. and her parents agreed to hospitalize her in a specialized inpatient ED department.

At admission, L. weighted 40 kg, her height was 1.63 m, and her body mass index (BMI) 15.4 kg/m^2^ (less than 5% BMI percentile for her age). She was diagnosed with AN purging type (AN-P). Her target weight was set at 53–56 kg, representing a BMI of 19.9–2, kg/m^2^ respectively. When she refused eating, nasogastric feeding was applied, and Fluoxetine 40 mg/day was initiated because of severe depression and eating-related obsessionality. During the first 3 weeks of inpatient treatment, L. was under constant 24-h supervision and was taken off responsibility for her T1DM treatment, being administered by the nursing staff. She made continuous efforts to influence her feeding program by slowing down the rate of the nasogastric feeding or tying up the feeding tube around her legs or neck. L. was asked to measure blood glucose levels with a sensor that was usually locked at the nursing room, in front of a nurse, which then would determine the amount of insulin required. Twice a day, she had to calibrate a sensor that measures glucose levels underneath the skin, providing dynamic glucose information and sending the data wirelessly to her parent's phone.

A few weeks after admission, following the resumption of oral feeding. L. started confronting new challenges. She had difficulty completing her meals, tried to hide food in her clothes or in her glucose sensor. Later, non-ED obsessional behaviors emerged: L. refused sitting on certain chairs or touch the pillows on a sofa, and separated the clothes in her closet with papers. A diagnosis of obsessive-compulsive disorder (OCD) was added. L.'s obsessional thinking also involved internal “rules” regarding her glucose levels, followed by repeated measures of glucose as part of related compulsions. Fluoxetine dosage was raised to 60 mg/day, with only a partial response of her OCD symptoms.

During her psychotherapy sessions, L. slowly began to address emotionally charged issues, revealing a long-standing body image disturbance that started already at the age of six. It seemed that the obsessional occupation with nutrition, weight, and appearance avoided her from confronting with T1DM-related difficulties, i.e., the strain caused by her highly intense dietary regimen, insulin treatment fidelity and family conflicts.

L. continued to restrict caloric intake, and started to omit insulin, or dilute her insulin with water during her visits home. She resumed nasogastric feeding and constant 24 hr supervision. Soon thereafter, L. disclosed to the staff about irritable suicidal thoughts, accompanied by non-suicidal self-injurious behaviors (NSSIs), laxative use and skin picking behavior. In the following weeks, she started disclosing her self-destructive behaviors throughout inpatient treatment: poor compliance with her medications, laxative pills hidden in her room together with shaving blades for cutting herself, and another glucose sensor in which she used to take blood samples directly from her self-injury wounds. During her home visits, episodes of hypoglycemia returned. In treatment, she described a desire for low glucose levels, feeling excited from putting her life in danger, and being disappointed that the hypoglycemic episodes only rarely led to unconsciousness.

As she kept gaining weight, L. expressed more and more depression, and wrote farewell letters to some staff members. She described self-infliction and insulin abuse as a means to “transfer pain from the mind to the body”, making her focus on nothing else but the cut or hypoglycemia.

Treatment with Fluoxetine was replaced with Venlafaxine 225 mg, and Quetiapine XR 150 mg was added, as an augmentation therapy for her depression. Gradually, L. started to cooperate with the diabetic program, and her mood stabilized. She began administering herself her insulin injections, and urine and blood glucose levels indicated that her diabetes was under good control. A year after her admission, L. was discharged to a day-care program. She took full responsibility for her diabetes treatment and avoided from NSSIs. Nevertheless, she still refused to tell her parents about her daily blood glucose levels, did not let them watch her calibrate her sensor, and rejected to eat her meals under their supervision.

Surprisingly, her HbA1c levels began to be much lower than the expected 4.8% norm. On her next visit to the endocrinology clinic, her sensor was taken for investigation, exposing multiple unreported episodes of hypoglycemia during the past 2 months. Her glucose levels were as low as 25 mg/dl, explaining her low HbA1c levels. As supervision was tightened once again, L. reacted with repeated caloric restriction. A short time later, she informed the staff about the resurgence of vomiting and laxative abuse and was re-hospitalized. She is currently still in inpatient treatment, with overall lack of cooperation, necessitating once again strict supervision.

#### Comment

L. is a 17.8 years old girl encountering multiple psychiatric and medical complications - AN, social anxiety disorder, depression, OCD, celiac disease, and T1DM. As highlighted in this case, youngsters with T1DM show considerable difficulties in handling their illness (50) and experience a multitude of psychiatric problems, likely interfering with the course of T1DM and with its treatment (51).

The picture becomes even more complicated in T1DM adolescents with comorbid EDs. Body image disturbances may increase the risk for a host of maladaptive weight-reduction behaviors. These may lead to lack of control over glucose levels, hence recurrent hypoglycemia, hyperglycemia, and unstable HbAIC levels (52).

As shown in the present case, intentional inappropriate handling of insulin administration to prevent insulin-related weight gain may occur in adolescents with comorbid T1DM and EDs (52). Recurrent insulin omission may be associated with diabetic ketoacidosis, whereas intentional insulin overdosing following high-calorie meals may induce hypoglycemia. If both insulin omission and insulin overdosing occur in the same ED patient because of faulty ED-related behaviors and cognitions, a vicious cycle of alternating ketoacidosis and hypoglycemia represents a particularly high-risk condition (51, 53). Alternatively, as in the present case, intentional insulin overdosing may serve as a destructive anxiety-reducing behavior in stressful situations.

In this patient's complex characterological and psychosocial constellation, the bidirectional insulin abuse likely involves multiple motives, in addition to weight reduction and body-image related motivations. L. associated her intentional insulin abuse with a wish to die (see her suicide letters) (54), or alternatively with an indifference to whether she would live or die, making this behavior highly life-endangering (53).

Third, intentional faulty insulin handling can serve as a means of communication to significant others (in the present case L.'s parents), when more adaptive communication means are lacking (37). In this respect, it can be regarded as an idiom of distress., i.e., as a way for this non-talking girl to express and communicate her distress, by repeatedly endangering her life (55).

Fourth, like many other maladaptive behaviors in AN, including NSSIs, intentional insulin abuse can serve to reduce mental pain by increasing psychic numbness, or alternatively, by conveying unspoken emotions. Last, the likelihood of insulin-overdose hypoglycemia for feeling “high” has been reported also elsewhere (53). Such a complex constellation in the face of conflictual motivations (L. did agree to be hospitalized) requires long-term inpatient treatment with recurrent hospitalizations, with questionable long-term outcome.

### Case 2: Psychogenic Polydipsia in a Patient With Anorexia Nervosa

S. is a 24-year-old single woman from a Jewish Ultra-orthodox background. She was accepted for treatment in an outpatient eating disorder (ED) service at the age of 22 because of a DSM-5 (1) diagnosis of AN-R.

S. is the second of four children. Her father is overweight, diagnosed with obsessive-compulsive disorder. Her older sister is diagnosed with binge/purge type ED.

Her development in younger years was uneventful, and she had no significant medical disturbances. She was always described as a shy child, with no friends, and as an average student. She was always on the lean side, but no disordered eating behaviors and preoccupations were reported.

After finishing high-school, she began studying in a seminar, where, for the first time, she was living outside of her home. She had no basic experience of cooking, and gradually started to eat less, at the beginning not because AN-related reasons. She gradually lost weight from 46 kg to 38 kg. Her height was 1.64 m. When this condition continued, she was referred to our outpatient clinic at the age of 22. Her menses ceased several months before admission. She was socially secluded, although living with other girls in her apartment, and was not interested to start with the process of “schiduchim”—getting acquainted with men for marital purposes.

On admission to outpatient service she reported of no wish to weigh more, or less, than her current weight. She was not concerned with her low weight (BMI-14.2 kgs/m^2^) or with the cessation of her menses. During outpatient treatment, no change occurred in her condition. Therefore, she was offered inpatient treatment, and, contrary to the therapists' expectations, agreed.

Her physical and laboratory examinations on admission were all within normal ranges. During inpatient treatment she gradually gained weight, until reaching the weight of 51 kg. (BMI-19 kg/m^2^), with gradual return of her menses. In contrast to the constant indifference and lack of emotional engagement during outpatient treatment, she became increasingly depressed, anxious and agitated, in inpatient treatment, with severe emotional dysregulation, and occasional suicidal preoccupations.

Psychotropic medications have been prescribed for the first time in inpatient treatment. She has been treated with Fluoxetine, Sertraline, Duloxetine, Aripiprazole, and Clotiapine, which reduced her anxiety and dysregulation, but had only minimal effect on her depression. Currently she receives Citalopram 40 mg/day, with some antidepressive effect, and Ritalin LA 30 mg/day because of attention deficit hyperactivity disorder.

During inpatient treatment, S. reported of starting to use laxatives, because of feeling bloated and distressed with her increase food intake. Later, she also admitted of self-induced vomiting for similar reasons. These behaviors were not present before inpatient treatment. Eventually these purging behaviors ceased following adequate supervision in the department and not allowing her to leave it.

The course of treatment seemed uneventful in the next weeks, until, in a routine examination before weighing, her urine concentration was unexpectedly very low (1.000, normal range in our laboratory is defined as 1.015–1.030). In a subsequent examination, it was still low (1.003), and she also presented with severe hyponatremia [serum sodium level of 120 meq/L (32), where normal values in our laboratory are 135–145 meq/L]. Physical examination and all other laboratory tests were normal.

In contrast with her cooperation in reducing laxative use and self-induced vomiting, S claimed that she was not drinking large amounts, although the staff noticed a recent increase in water consumption. Against her will, she was supervised for her water consumption, showing that she was drinking daily around 4 L, double the average amount required in the Israeli climate.

She was put on a strict water consumption regimen, and her urine concentration and sodium values gradually stabilized. Thereafter, she stated going out again from the department, with occasional reduction in her urine and serum sodium concentrations, requiring supervision again.

The association of her serum sodium and urine concentration levels with the extent of water consumption during supervision, likely precluded the diagnosis of inappropriate antidiuretic hormone hypersecretion as the cause of hyponatremia. This was likely the case also with respect to her use of psychotropic medications. Theoretically, antidepressant and antipsychotic medications may induce hyponatremia by excess water intake provoked by a sensation of dryness of mouth from anticholinergic adverse effects (tricyclic antidepressants and neuroleptics), as well as from inappropriate ADH secretion due to selective serotonin reuptake inhibitors use (56).

One day, without the staff knowledge, she went to s swimming pool, and had what seemed like a loss of consciousness while being in the water. People who put her out of the water were not sure whether she was convulsing or not, but she bit her lips, and was bleeding from her mouth. She did not pass urine or stool and was not judged to be drowned.

S. was brought to the emergency room, while already being awake but somewhat confused. She was disoriented to time. Her speech was slurred, and she had no memory of what has happened to her. Her last memory was of entering the pool's surroundings, not of being in the water.

Physical and neurological examination performed in the emergency room, including fever, pulse, and blood pressure, was normal. She had further no evidence of subcutaneous edema. Laboratory examinations were within normal ranges, except for sodium level of 119 meq/L, and urine concentration level of 1.000. Chest-ray examination was intact. Electrocardiogram and later Holter electrocardiogram showed no evidence of arrythmias. EEG revealed diffuse slow wave activity, with no localization, later returning to normal. Later Holter EEG was normal. MRI showed no evidence of cerebral edema, space occupying lesion or of past/recent trauma. She was diagnosed with loss of consciousness resulting from water intoxication and hyponatremia, requiring only ongoing supervision.

Upon her return to the department, S. was put again on constant supervision. Upon re-stabilization, she was put on plan where she was supervised for her urine concentration every other day, and had a bi-weekly check of her serum sodium levels. If urine concentration was normal, she was put on a daycare regimen. If, not, she was re-hospitalized until her next urine concentration check.

On the one hand, this plan increased her motivation to cooperate with the water consumption plan, and her serum sodium and urine concentration levels were mostly normal. On the other hand, when likely having no at-risk behaviors to counteract her cooperation with weight gain and eating (she chose to stay in the department although she could leave it any minute), S. became more depressed and frustrated. Although having a place to go after her release (her previous apartment), and although having a job plan and a financial support (learning to become a professional secretary), S. felt she had no real goal in her life, and began questioning about the meaning of her living. It seemed that once she became relatively asymptomatic with her eating, her existential depression and sense of futility increased. Currently, psychotherapy focused mainly on dealing with these issues under day-center placement. It is of note, that S. hardly related to the event at the pool. She was not concerned with the possibility that she could have died, and she did not relate to the event as an attempted suicide, but was afraid of possible resulting medical complications.

#### Comment

The process of events occurring in the life and treatment of S. highlights several important points. Although living within her family and being part of her community, S. always felt that she was keeping herself on the verge of living, with no real inner direction. She did things feeling that this was expected form her and left her home unprepared. The reduction of eating in the beginning was not intentional, but rather reflected her overall lack of acquaintance of and indifference to her own needs (22), thus not taking any notice that she was not eating for long hours.

S. agreed to outpatient treatment similar to her previous matter of-fact attitude, as another meaningless station in her life. It was not surprising that it had no effect on her illness. Her agreement to inpatient treatment was however, highly different. On the one hand, she cooperated with the treatment plan, and did not leave the department, although having the possibility to do so. On the other hand, she began to be involved in constant at-risk behaviors, developing from laxative use and self-induced vomiting to psychogenic polydipsia. These behaviors reflected mainly her inability to live with the emotions and physical sensations induced by her increased eating and weight. Typical to patients with AN, she was unable to deal with these distressing feelings and sensations and communicate them to others (16, 21), but rather to act out on them.

The indifference of S. to the almost fatal results occurring in the swimming pool, while partly associated with her not remembering the event, mostly reflected her lack of interest as to what might have happened with her immediate life (23). Such an indifferent attitude, leading to recurrence of water intoxication following hospitalization because of grave medical complications of this behavior, has been reported in AN patients also elsewhere (24). This indifference likely reflects the inability of the AN patient to look at herself from the outside and grasp what she is really doing to herself, i.e., deficiencies in reflective mentalization and theory of mind capabilities (57, 58).

Despite these reservations, the agreement of S. to cooperate with the treatment plan, while simultaneously actively involved in at-risk behaviors opposing it, was a change in her overall aloofness. It could, however, take place only in the strictly supervised, organized, predictable and safe inpatient environment. There, likely for the first time, she was able not to block her emotions, as often occurs in patients with AN (59), but rather to really feel. The overwhelming flooding of unclear, unknown, and highly distressing emotions likely increased her at-risk behaviors (59). Nonetheless, her connectedness with her emotions (22) might increase, in due time, the chance of improving with psychotherapy in the flexible and safe environment of moving from daycare to inpatient treatment as required.

### Case 3: Laxative Abuse Leading to Rectal Prolapse

N, is in her late thirties, single, unemployed, living with her parents, despite a complicated relationship with them. She reports domestic violence and feelings of fear, alongside dependence, and excessive closeness. Her ED appeared when she was 14. She began restricting her eating and vomiting and became underweight (minimal BMI was 14.7 kg/m^2^). Diagnosed with AN-P (1), she was hospitalized in a specialized ED unit. After discharge, her condition improved, but she still experienced occasional patterns of fasting, bingeing, and vomiting, meeting DSM 5 (1) criteria for BN.

Following graduation from high school, she began working; she then suffered of sexual abuse, perpetrated by her supervisor at work. Since then, her ED symptoms worsened considerably. She developed bingeing, vomiting, and use of large quantities of laxatives daily, leading to two additional hospitalizations in a specialized ED department; another hospitalization was in a psychiatric department following a suicide attempt using pills. Over the years, she also became addicted to alcohol.

Approximately 2 years before her current hospitalization, she completed an addiction treatment program and continued to attend Alcoholics Anonymous meetings. She was re-hospitalized because of severe deterioration in her ED symptoms, including massive laxative use.

N. was hospitalized with a BMI of 18.4 kg/m^2^. Psychopharmacotherapy included Topiramate 200 mg/day, Quetiapine 500 mg/day, Fluoxetine 60 mg/day, and Clonazepam 1.5 mg/day. At admission, her blood potassium level was 3.1 mmol/L, gradually rising to normal levels, likely reflecting discontinuation of laxatives use and vomiting.

After 2 months of inpatient treatment, hypokalemia was observed again (potassium levels decreased to 2.9 mmol/L). When questioned, the patient denied reverting to laxative use or vomiting, and her reports of diarrhea accompanied by general ill feeling were interpreted at that time as being of viral origin. However, shortly thereafter, she reported that when sitting on the toilet, she felt organs protruding from her anus. She was extremely frightened when a substantial bowel segment protruded, and had difficulty reinserting it. N. was examined by a surgeon, who diagnosed rectal prolapse. Surgery was recommended. It was explained to the patient that renewed vomiting and laxative use after surgery would endanger her, and that the surgery would only be performed if she could unequivocally commit to avoiding any return to vomiting and laxative use. in her psychotherapy sessions, she expressed full understanding of these risks and motivation for treatment. The conclusion of department's staff was of a high probability that she could control her symptoms. N. was transferred to the surgical department, where she had undergone laparoscopic sigmoidectomy with rectopexy.

Ten days after surgery, she was readmitted to the ED unit, where she rapidly resumed regular nutrition, and attained normal weight. She then began to build a rehabilitation plan, including transition to an ED residential rehabilitation center. Soon thereafter, her blood tests indicated hypokalemia again, but in consultation with the surgeon, this result was attributed to surgery-related watery discharge. However, near to her discharge from inpatient treatment, laxatives that she had hidden, were discovered in her possession. The patient admitted that 2 weeks after surgery, she started using laxatives again. She related this abuse to the distress she felt after surgery, in the anticipation of her soon-to-come discharge. She was referred for laxative addiction treatment in a group setting following her discharge.

#### Comment

N. suffers from a severe and enduring B/P-type ED, exhibiting a combination of restricting, bingeing, self-induced vomiting and laxative abuse, alongside additional alcohol addiction. Similar to our patient, other studies have also shown that the combined presence of vomiting and laxatives/diuretics misuse (multiple purging behaviors) is associated with greater ED severity, more comorbidity with depression and post-traumatic stress disorder (PTSD), greater use of alcohol and drugs, and a high incidence of sexual or other traumatic events, usually predating the development of the ED and the comorbid substance abuse (60, 61).

The presence of multiple purging behaviors may enable the patient to dissociate from and ignore her overwhelming trauma-related thoughts and emotions, thus potentially cleansing herself of the experience (62). From a different perspective, the lack of emotional regulation typical of such patients (16, 21), may be related also to problematic early life attachment relations (63). N. has constantly turned during her life course to the abuse of various substances and to a multitude of ED symptoms to soothe and regulate her turbulent emotions, as well as to turbulent abusive relationships, almost always ending in explosive fights and recurrent abandonments.

Bromberg (62) points to the damage to trust and to the belief in the possibility of repair within a therapeutic relationship in patients with EDs who have experienced early developmental disturbances and later traumatic harm. N. developed ED symptoms by the age of 14. Her fragile self-structure was insufficient to cope with the sexual trauma she later suffered, at the age of 20, leading to escalation of her multiple symptoms—restricting, bingeing, vomiting, laxative abuse, alcohol addiction, depression, and suicidality. Within the supportive environment of the inpatient setting, N. was able to use psychotherapy to identify the way in which she turned to objects, such as laxative abuse, for emotional regulation instead of to human relations (22). However, when she had to be separated from the therapeutic support she had only started to learn to rely on, her almost automatic, impulsive reliance on objects (laxatives) to regulate her anxiety re-emerged (8, 16, 17, 22). This led to renewed laxative abuse, despite her understanding of the high risk involved in it and the possible disruption of the results of her surgery (62, 64).

### Case 4: Pregnancy and Anorexia—Which Has the Upper Hand?

M. aged 23, has been suffering from AN-B/P since the age of 12. Born to an Ultra-Orthodox Jewish family and married at the age of 20, M. tried to conceive in the first 2 years of her marriage and had one natural miscarriage before succeeding, following fertility treatments. She kept the fertility treatment a secret so as not to impair with her sisters' marriage prospects.

Her ED was prominent throughout her upbringing. M. recalls how people would complement her for her slim figure. Her low weight gained her special attention among her family members, as well as constant worries by her mother, who kept certain items in the family refrigerator reserved solely for M., and absolve her from certain house chores. Her husband was described as supportive, but unaware of her illness.

At 23 weeks of gestation, M. was referred to the emergency room after having fallen in the street, complaining of abdominal pressure. Her BMI at that time was 17. She had virtually not gained any weight during pregnancy, while reporting of self-induced vomiting. The fetus was 8 weeks delayed in development. M had at that time anemia, hypoglycemia, and protein C deficiency. After 2 days of failed attempts to feed M. at the high-risk pregnancy unit, she consented to be hospitalized in a specialized inpatient ED department.

M's treatment plan included complete bed rest, nasogastric tube feeding and cardiac monitoring. M reported she was happy to begin psychotherapy. In the sessions she disclosed of multiple weight-reduction behaviors during her pregnancy, for example going up and down the stairs for 2 hr daily, followed by abdominal exercises.

In the second week of inpatient treatment, M gained 1 kg. She reported trying to view her weight gain as part and parcel with the keeping of her pregnancy, but this understanding did not alleviate the difficulty and fear of gaining weight. She feared the weight gain and the related post-natal struggle to lose weight. On the one hand, M described her body as “good”, insofar as it carried a baby, to whom she has much to provide. She did not want to harm the baby. At the same time, however, she felt huge. She touched her belly and asked whether her fat percentage could be checked.

During the third week of inpatient treatment M was caught trying to spill the nasogastric tube content down the toilet. She expressed her wish to terminate the hospitalization. The staff explained her the severe consequences to the development of the fetus that could ensue from this decision. M. agreed to continue hospitalization only after her husband, who had become involved in her treatment, convinced her to remain in the hospital. In the following psychotherapy session, she described a feeling of loneliness, and that the staff were trying “to shake her” not to think of herself, but to change her priorities to caring for the fetus. This, however, evoked in her a wish to restrict eating, to vomit, to prove the importance of her own needs, and to convey the suffering she was experiencing.

M. confides to her therapist that she fears also her criticism. The therapist admits to herself that there is some truth to her patient's fear. She recalls her dismay upon hearing of her patient's abdominal exercises and intense physical activity she has undertaken during her pregnancy. When being informed of the delay in the development of the fetus, the therapist finds herself angry at her patient, and cannot stop thinking about the baby. She is alarmed of the intensity of her negative feelings towards her patient and expresses this fear in her supervision.

In the following 2 weeks, M. becomes more and more upset. It appears that despite the fear of not feeding her fetus properly, the fear of weight gain overwhelms everything. At 28 weeks gestation, M. decides to discharge herself against medical advice. This time even her family and husband are unable to convince her to stay in treatment. M has gained 2 kg during her 5-weeks hospitalization.

M gave birth at 34 weeks, needing a blood transfusion during delivery. Her hemoglobin level decreased to 6.9 g/L. The baby weighed 1.6 kg at birth, with an Apgar score of 4, and evidence of microcephaly.

#### Comment

Women suffering from AN may refuse to abandon the dream of motherhood, and some believe in and achieve recovery with the child they are bearing (48, 65–69). Nonetheless, this dream can be easily shattered in the face of bodily and hormonal changes, putting the pregnant woman with AN against stressful dramatic challenges: rapid weight gain, expansion of her bodily contour, rounding of her breasts, and an overall feeling of lack of control over her body. Research frequently points out to the dangerous coexistence of the two, whereby pregnant women with EDs show greater risk of miscarriage, delayed intrauterine growth, premature labor, forced caesarian deliveries, low birth weight, and abnormally small head circumference at birth (67, 69–76).

In this respect, pregnancy in women with an ED is a high-risk pregnancy (49). In the present case, M. has not sought for any help for her ED, until 23 weeks of gestation. Thus, the low hemoglobin levels during the first trimester have been likely associated with the intrauterine growth delay and low weight at birth (77).

The present case demonstrates the danger of pregnancy becoming unbearable for a woman suffering from active AN, as it may stand against the core goal of the ED - having a perfectly thin body. The conflict of M. between her need to care for the fetus and her fear of the change pregnancy has induced in her body and in her own well-being, has been evident throughout pregnancy. Unfortunately, as in the present case, the ED has prevailed.

The psychological challenges of a woman with AN during pregnancy can be challenging also to her therapist, particularly in young female therapists who are themselves mothers or future to be mothers. Sometimes, the fears of the pregnant AN patient may not be apparent to the therapist, who is torn between the caring for the needs of her patient—the mother vs. the needs of the fetus. In the case of M., the therapist has been overwhelmed with strong emotions of concern for the future-to-be baby's wellbeing, with accompanying anger toward the abusive mother. Thus, the therapist has become a mother who fails to nurture her own child, her patient.

Medical and psychological support for pregnant women with severe EDs should be based on a multi-professional team, including experts from both the gynecological and ED professions. Hospitalization in specific ED facilities should be an option in severe cases such as ours. The present case exemplifies the great difficulty in dealing with pregnant ED women who refusing treatment even in an environment that is safe for them and their babies. In these cases, it is advisable to seek support from people that are most meaningful to the patient, including the patient's spouse, family, and her community at large (in the present case a religious authority could have been an option). Should the patient release herself from inpatient treatment against medical advice, it is important to continue with physical and psychological monitoring in specialized ambulatory community treatment environments to provide support for the mother and the baby, during and after birth. Compulsory treatment in such cases is not a possibility under the Israeli law.

## Discussion

The present article described four women with EDs who were engaged in extreme self-inflicted behaviors that endangered their life and health (all cases) and that of their future-to-be baby (case 4). They were of different ages, from 17.8 to the late 30^th^, had engaged in different types of extreme at-risk behaviors, and were treated in different specialized ED centers in Israel.

These specific cases were chosen because they reflected the broad spectrum of severe medical complications associated with their ED-related at-risk behaviors. These medical complications, alongside the patients' severe ED symptomatology and comorbid psychiatric disturbances, required prolonged, often repeated, hospitalizations in medical and/or specific ED departments. The patients mostly did not comply with the many medical and psychological opportunities they were offered. This required particularly fine-tuning, determination and patience from the multi-professional treatment providers. Unfortunately, none of the patients showed any inclination toward remission of their ED. Hence, all were at risk to continue with their life-endangering behaviors.

Several factors were common to all or most patients:

1. All patients were diagnosed with AN with B/P pathology (case 2 switched from restricting to B/P pathology during inpatient treatment). Second, three patients (cases 1–3) had evidence of more than one purging behavior, i.e., both self-induced vomiting and laxative abuse; this suggests the likelihood of considerable impulsivity associated with multiple purging presentation (8, 60, 78). Third, several patients had evidence of non-ED-related impulsive behaviors (case 1: oppositional behavior, NSSIs; case 2: attention deficit hyperactivity disorder; case 3: alcohol addiction). Altogether, although impulsivity was not directly assessed, the extreme self-endangering behaviors in our cases were likely carried out in the context of severe impulsivity.
2. In addition to impulsivity, most patients suffered from other comorbidities and from multiple vulnerabilities. Case [1] was diagnosed with social anxiety disorder, depression, and OCD. She was introverted and shy, had communication problems as a child and almost no friends, and her relations with her parents were highly conflictual. At times she was highly suicidal. In addition to the ED, she also suffered from celiac disease and T1DM. Case [2] suffered during inpatient treatment from depression, anxiety, and occasional “giving-up”-related suicidal preoccupations. Case [3] had abusive, violent, overly-dependent relations with her parents, as well as undergoing sexual trauma as a young adult. Altogether, as demonstrated in our patients, extreme at-risk behaviors in patients with AN seem to occur in the context of a long-standing extremely harmful background. In support of this contention, it has been repeatedly found that sexual trauma in patients with EDs is associated with the occurrence of multiple purging behaviors, comorbid depression, PTSD, substance abuse, and elevated suicide risk (60, 61, 79)
3. In most patients, the extreme at-risk behaviors developed on a background of long-standing severe AN, often requiring multiple hospitalizations (case 3): duration from onset to around 4 years in cases [2] and [3], and 9 years in case 4. Most, although not all, extreme at-risk behaviors were carried out in the purpose of weight reduction and body image disturbances. In case [1], insulin omission was used to lose weight, in addition to restricting and purging behaviors. In case [2], although the patient claimed that her excessive water consumption was not done to reduce her eating, it nevertheless appeared after the cessation of vomiting and laxative misuse. In cases [3] and [4], the grave consequences (rectal prolapse and early birth, respectively), were the direct result of multiple problematic eating-related behaviors.
4. Nonetheless, the most important finding related to these case descriptions was that multiple co-occurring non-ED motivations and faulty emotional handling, may lie at the base of the extreme endangering behaviors described here, above and beyond ED-related factors. In general, although most patients were suicidal (cases 1–3), except for patient [1], who directly attached her faulty insulin handling with her wish to die, or with an indifference to whether she would live or die (53, 54), none of the other patients attached their severe at-risk behaviors with suicide wishes. Second, whereas the motivations inherent in the severe self-destructive acts here were like those found in NSSIs (8, 16–18), in contrast to NSSIs, the symptoms caused by these behaviors almost always involved massive medical disturbances.
5. The problems and motivations underlying these extreme behaviors included: 1.) difficulties in emotional regulation and expression, and extreme unexpected moves from emotional blocking to emotional flooding [(16, 21, 59); cases 1–3]. The handling of these conditions likely required extreme self-destructive acts for anxiety reduction and self-soothing, and for avoiding contact with overwhelming emotions (case 3), mental pain (case 1), or depression [(case 2) (62); 2.)] as an idiom of distress, to indirectly communicate one's suffering to the environment in individuals with communication problems [all cases to some extent; (55), and 3.] as potentially related to early maltreatment, leading to problematic attachment relationships with significant others throughout life [cases 1, 3; (63)], specifically when separation is imminent upon the improvement of the ED (case 3).
6. Other motivations may also potentially account for the extreme at-risk behaviors described here: In case [1], the use of insulin overdose to create hypoglycemic episodes to feel “high” and excited from endangering her life (53). In case [4], the ongoing multiple faulty eating-related behaviors, signify the unresolved conflicts of caring for oneself and fostering one's own space and self-identity vs. being a good mother and caring for her future-to-be child.
7. In all cases, the patients seemed to be indifferent to the potentially harmful consequences of their repeated extreme self-endangering acts. This indifference might be associated with the motivations to the extreme behaviors described here. Specifically, it might reflect the tendency of patients with AN to deny the severity of their illness (1), that might be generalized to other medical disturbances. It might be further associated with the patients' deficient emotional, mentalization and theory of mind capacities [(21), 58.59], as well as their reduced attraction to life (25), when having no immediate or long-term fulfilling meaningful goals.
8. The treatment of all four cases required inpatient settings specializing in the treatment of severe long-standing EDs, as well as of severe self-endangering behaviors often requiring intensive medical interventions. The treatment in all centers described here is multidimensional and multi-professional. The patients receive a structured nutritional rehabilitation program, as well as multimodal individual, family and group interventions tailored for the treatment of the ED, comorbid disorders, and different psychosocial difficulties
9. At the beginning of hospitalization, individual psychotherapy in most centers was supportive, to assist the patients in in their nutritional rehabilitation, alongside cognitive behavioral therapy (CBT) and dialectical behavior therapy (DBT) elements as required. DBT was of particular relevance for emotionally dysregulated patients involved in impulsive life-endangering behaviors such as ours (80). At later stages, treatment included motivational and different types of psychodynamic psychotherapies. For the present cases, psychodynamic interventions were considered to compensate for the patients' long-standing emptiness, loneliness, frustration, lack of self-fulfillment, and severe enduring relational difficulties. These interventions were also considered to potentially enhance the developmental abilities of our patients in front of the challenges of discharge from inpatient treatment, which was highly difficult for them. Whereas adolescent patients usually return to their home, adult patients may receive rehabilitation interventions preparing them for referral to post-discharge ED-related rehabilitation centers. Unfortunately, all the cases described here, lacked the motivation and resources to be referred to such rehabilitation centers.

## Data Availability Statement

The datasets generated for this study are available on request to the corresponding author.

## Ethics Statement

Ethical review and approval was not required for the study on human participants in accordance with the local legislation and institutional requirements. Written informed consent to participate in this study was provided by the participants' legal guardian/next of kin.

## Author Contributions

All authors (DS, SK, II, TS, AT, MP, EW) contributed to the conception and design of the study. SK, II, TS, AT, and MP contributed the different case reports. DS and EW contributed the introduction and general remarks and were responsible for the organization of the article. All authors read and approved the final draft of this article.

## Conflict of Interest

The authors declare that the research was conducted in the absence of any commercial or financial relationships that could be construed as a potential conflict of interest.
